# An Account of a Safe and Efficacious Medicine in Sore Eyes and Eye-Lids

**Published:** 1782

**Authors:** 


VIII. *An account of a ſafe and efficacious medicine in ſore eyes and eye-lids. By* Thomas Dawſon, *M. D.* 8vo. Johnſon, *London*, 1782. 15 pages. 1s.

AFTER many fruitleſs inquiries concerning a medicine for diſeaſed eyes, which above fifty years ago was in the poſſeſſion of Dr. Thomas Nettleton of Halifax, in Yorkſhire, our author at length, in 1763, met with a near relation of his own, who having been a conſiderable ſufferer from ophthalmia, informed him ſhe never found ſo much benefit from any means uſed for her relief as from an ointment preſcribed for her by Dr. Nettleton himſelf, and with which, after his deceaſe, ſhe had at times been ſupplied by Dr. Key of Mancheſter, who had formerly been a pupil to that gentleman.

Well perſuaded of Dr. Key's benevolence, our author applied to him for the receipt, which he readily communicated, adding, at the ſame time, ſome uſeful hints relative to his own practice and experience, with reſpect to this medicine.

The

The Receipt is given in the following words from Dr. Key's letter on the ſubject:

℞. Butyr. ℥viij. Aq. Fort. Comp. Argent. Viv. ā ℥j. Camphor. ʒij. Butyro liquefacto et in coagulum denuo tendenti, injice argentum vivum in Aq. Forti ſolutum et Camphoram in olei olivarum ℥ij ſolutam diligenter agitans in mortario marmoreo donec refrixerint, ut f. ſ. a. Unguentum.

Dr. Key obſerves that when this has been long kept, it becomes friable ſo as to require a little more oil to be added to it; but that let it be ever ſo friable, it will eaſily ſoften by being held to the fire.

If the inflammation is violent Dr. Key recommends topical bleeding, and even ſcarification of the eye-lids previous to the uſe of the ointment; but if the inflammation is moderate, the patient, we are told, may begin with it without any previous bleeding. Cooling purges, however, are to be preſcribed.

For a film in the eye we are directed to ſoften the ointment by holding it to the fire, and then taking up ſome of it upon a fine camel-hair pencil, to draw it over the film twice a day.

